# Sex differences in IL‐6 responses to moderate‐intensity aerobic exercise are explained by cycling power output

**DOI:** 10.14814/phy2.70850

**Published:** 2026-04-03

**Authors:** Ena Yoshida, Harumi Hayashida, Tung Lin Lu, Yasuhiro Seki, Katsuhiko Suzuki

**Affiliations:** ^1^ Graduate School of Sport Sciences Waseda University Tokorozawa Saitama Japan; ^2^ Faculty of Sport Sciences Toin University of Yokohama Yokohama Kanagawa Japan; ^3^ Faculty of Sport Sciences Waseda University Tokorozawa Saitama Japan

**Keywords:** carbohydrate oxidation, d‐ROMs, exercise‐induced stress response, interleukin‐6, sex differences

## Abstract

This study characterized interleukin‐6 (IL‐6) and derivative‐reactive oxygen metabolites (d‐ROMs) responses to moderate‐intensity exercise in healthy adults and assessed whether sex differences were due to workload. Fifteen males (26.5 ± 2.9 years, BMI: 23.7 ± 3.1) and 14 females (25.7 ± 2.8 years, BMI: 21.2 ± 1.8 in the menstrual phase (MP) and 21.8 ± 2.1 in the luteal phase (LP)) completed 60 min of cycling at 65 ± 5% of predicted maximal heart rate. Power output was 42.8 ± 11.8 W (MP), 44.0 ± 14.1 W (LP), and 78.8 ± 26.9 W (males) (MP vs. males and LP vs. males: *p* < 0.001). IL‐6 and d‐ROMs were measured before (Pre), immediately after (Post0), and 60 min after exercise (Post60), and group × time effects were tested using linear mixed‐effects models. IL‐6 showed a group × time interaction. Males had greater IL‐6 responses than females at Post0 (MP vs. males: *p* < 0.001; LP vs. males: *p* < 0.05), but these differences were not significant after adjusting for power output. d‐ROMs showed no significant changes over time or by group. Power output correlated with carbohydrate oxidation. These findings suggest that, at the same relative intensity, sex differences in IL‐6 responses reflect workload.

## 1 INTRODUCTION

Exercise is a lifestyle habit recommended for a broad range of people, regardless of age, sex, or health status, and it plays an important role in the prevention and improvement of chronic diseases and psychosocial health (Bull et al., 2020; World Health Organization, 2020). However, there is considerable interindividual variability in the physiological responses to exercise, and the underlying biological factors contributing to this variability remain incompletely understood (Noone et al., 2024). In particular, sex differences have recently attracted growing attention as a factor that may determine differences in physiological responses during exercise and in training adaptations (Ansdell et al., 2020; Hunter & Senefeld, 2024). Nevertheless, because most research in medicine and sports science has historically been conducted predominantly in young, healthy males, evidence that adequately accounts for sex is still limited. Consequently, there is increasing concern that current standard training programs and guidelines may not necessarily be optimally effective or safely implemented when applied to females (Cowley et al., 2021; Elliott‐Sale et al., 2021). Therefore, quantifying sex differences in physiological responses to exercise and identifying the factors necessary for their interpretation is crucial for developing optimal exercise training methods for individuals.

Some of the health benefits of exercise can be explained by exercise‐induced transient physiological stress (Hackney, 2006; Mastorakos et al., 2005). The human body has the capacity for plasticity, whereby its functions are altered in response to the environment in order to maintain homeostasis, and defensive mechanisms are induced in response to the stress imposed by exercise. Repeated exposure to such stress is thought to enhance basal physiological function and confer greater tolerance to subsequent stressful stimuli (Ji et al., 2016; Petersen & Pedersen, 2005; Radak et al., 2008; Suzuki et al., 1999). Inflammation and oxidative stress are among the most representative markers of exercise‐induced stress and are considered important mediators of its effects. Among these, the inflammatory cytokine interleukin‐6 (IL‐6) shows a marked increase in circulating levels during acute exercise (Steensberg et al., 2000). IL‐6 helps regulate whole‐body energy supply by stimulating hepatic gluconeogenesis and promoting glucose uptake in skeletal muscle (Febbraio et al., 2004; van Hall et al., 2003). It also exerts anti‐inflammatory effects by inducing interleukin‐10 (IL‐10) and suppressing tumor necrosis factor‐α (TNF‐α), thereby attenuating systemic inflammation (Starkie et al., 2003; Steensberg et al., 2003). These actions contribute to the maintenance of health, in part by improving insulin sensitivity and suppressing chronic inflammation. In addition, the exercise‐induced increase in oxygen consumption and repeated muscle contractions enhance the production of reactive oxygen species (ROS) (Powers & Jackson, 2008). Although excessive ROS generation results in oxidative stress and causes oxidative damage to tissues, a moderate increase in oxidative stress is thought to act as a hormetic stimulus that, via activation of redox‐sensitive signaling pathways such as nuclear factor erythroid 2–related factor 2 (Nrf2) and peroxisome proliferator‐activated receptor γ coactivator‐1α (PGC‐1α), induces the expression of antioxidant enzymes and genes involved in mitochondrial biogenesis, thereby improving skeletal muscle mitochondrial oxidative capacity with training and reducing the risk of chronic diseases (Gomez‐Cabrera et al., 2008; Ji et al., 2006; Powers & Jackson, 2008; Silveira et al., 2006).

Regarding sex differences in the IL‐6 response to exercise, conclusions are inconsistent. Some studies report that females exhibit a greater IL‐6 response than males 60 min after maximal exercise (Edwards et al., 2006). Conversely, other studies indicate that blood IL‐6 levels rise significantly after competitive marathon running in both males and females, with the magnitude of change not influenced by sex (Nieman et al., 2001). Furthermore, reports suggest timing differences in peak IL‐6 levels: after a single bout of total body resistance exercise, peak levels occur 1 h later in males and 4 h later in females (Benini et al., 2015). Similarly, regarding sex differences in the oxidative stress response to exercise, while some reports indicate that changes in oxidative stress markers after running exercise are generally similar between males and females (Goldfarb et al., 2007; Pepe et al., 2009), other studies show that after a single bout of eccentric and concentric running, oxidative stress increases in females but remains unchanged in males (Wiecek et al., 2017). In addition, reports indicate that after endurance exercise, males exhibit a greater antioxidant response and suppression of oxidative stress compared to females (Ginsburg et al., 2001). Furthermore, oxidative stress results can be influenced by the specific markers used (Kawamura & Muraoka, 2018). One oxidative stress assessment indicator, derivative‐reactive oxygen metabolites (d‐ROMs), reflects systemic oxidative stress status by quantifying circulating hydroperoxides (Costantini, 2016; Seki, 2009). This indicator is suitable for repeated‐measures studies tracking time‐series changes induced by exercise, as it allows rapid measurement using minimal samples (Hayashi et al., 2007; Ikeda et al., 2023; Takahashi et al., 2015). Furthermore, it has been demonstrated that the menstrual cycle influences IL‐6 and d‐ROMs values in females (Eagan et al., 2021; Matsuda et al., 2020; Yoshida et al., 2025), making control for the menstrual cycle crucial when examining sex differences.

Exercise‐induced IL‐6 and oxidative stress responses are closely related to the amount of skeletal muscle recruited and the metabolic demand during exercise (Fischer, 2006; Helge et al., 2003; Steensberg et al., 2000; Wiecek et al., 2017). Males and females differ significantly in skeletal muscle mass and maximal oxygen uptake. Therefore, even when exercise is prescribed at the same relative intensity, differences in the absolute amount of skeletal muscle recruited and metabolic rate during exercise may occur, potentially leading to differences in inflammatory and oxidative stress responses. However, existing studies have not statistically adjusted for these factors or evaluated their contributions. Consequently, it remains unclear whether observed or unobserved differences stem from biological sex itself or are secondary effects mediated by these physiological differences.

Based on this background, we had males and females perform a bout of moderate‐intensity aerobic exercise at the same relative intensity as would be recommended for the general population, and used IL‐6 and d‐ROMs as markers. Furthermore, we aimed to conduct an exploratory evaluation of whether these response differences were associated with factors such as skeletal muscle mass (SMM) and metabolic demand during exercise.

## 2 MATERIALS AND METHODS

### 2.1 Ethical approval

This study was conducted in accordance with the Declaration of Helsinki and was approved by the Ethics Committee of Waseda University (approval number 2024‐341). Written informed consent was obtained from all participants prior to their participation in the study.

### 2.2 Participants and exercise testing procedures

Healthy nonathlete males and females aged 23–35 years without chronic disease or a smoking habit were recruited for this study. Fifteen males and 14 females participated in this study. Females had regular menstrual cycles (25–38 days). Female participants provided information on their recent menstrual cycles, and detailed dates for three cycles were obtained for each participant. Males completed a single exercise trial on an arbitrary day, whereas females performed two exercise trials, one in the menstrual phase (MP) and one in the luteal phase (LP), in random order. The MP was defined as days 1–5 from the onset of menstruation, during which menstrual bleeding persisted. The LP was defined as a period corresponding to the latter half of the cycle, based on each participant's own cycle records. In this study, the MP, representing a low‐hormone environment, and the LP, characterized by a contrasting hormonal profile, were selected as the measurement periods. The MP is a physiologically important period for evaluating exercise‐induced inflammatory and oxidative stress responses.

The exercise test was performed between 9:00 and 13:00. Participants were instructed to refrain from vigorous physical activity and alcohol consumption for 24 h before the test and to abstain from any food or drink other than water for 12 h before the test. On the test day, height and body composition were measured using a multifrequency bioelectrical impedance analyzer (InBody 720, InBody Co., Ltd., Seoul, Korea). The tests were conducted in a climate‐controlled chamber maintained at a temperature of 20°C and a humidity of 50%, and participants performed 60 min of aerobic exercise on a cycle ergometer (AEROBIKE 75XL III, Combi Wellness Co., Ltd., Tokyo, Japan). Exercise intensity was set at 65% (maintained within ±5%) of the age‐predicted maximal heart rate, calculated using the Equation 208 − (0.7 × age) (Tanaka et al., 2001), and the wattage of the cycle ergometer was adjusted while heart rate was monitored in real time using a heart rate monitor (H10 N, Polar Electro Oy, Kempele, Finland) attached to the chest. Participants were instructed to maintain a pedal cadence of 60 revolutions per minute throughout the test. All wattage adjustments were performed by the same person in all tests. During the exercise, ratings of perceived exertion (RPE) for the legs and breathing were obtained from the participants every 10 min and recorded.

The composition of expired gas during exercise was measured in 30‐s averages using an automated gas analyzer (Aero Monitor AE‐310S, Minato Medical Science Co., Ltd., Osaka, Japan). From these data, carbohydrate was calculated according to the following previously reported Equations (Péronnet & Massicotte, 1991; Takken et al., 2005).

$$\begin{array}{c} \text{Carbohydrate oxidation}\;\left(g/\min\right)\\ =4.585\times {\mathrm{VCO}}_{2}\;\left(L/\min\right)-3.226\times {\mathrm{VO}}_{2}\;\left(L/\min\right) \end{array}$$

### 2.3 Blood collection and analysis

Blood sampling was performed at three time points: before exercise (Pre), immediately after exercise (Post0), and 60 min after exercise (Post60). The collected blood was dispensed into vacuum blood collection tubes containing a serum‐separating agent. Serum samples were allowed to stand at room temperature for 30 min and then centrifuged at 3000 rpm for 10 min, after which they were stored at −80°C until analysis. IL‐6 concentrations were measured in serum using a human IL‐6 ELISA kit (Quantikine HS600C, R&D Systems, Minneapolis, MN, USA). d‐ROMs were measured in serum using a commercial kit (d‐ROMs Test Kit, DI‐003b, Wismerll Co., Ltd., Tokyo, Japan). The d‐ROMs test is a colorimetric method that measures organic hydroperoxides present in serum to indirectly assess systemic oxidative stress. Organic hydroperoxides are primary oxidation products generated by the oxidation of lipids, proteins, and nucleic acids. This indicator reflects the total amount of organic hydroperoxides in circulating blood and does not identify specific sites of production. The values obtained reflect the degree of oxidative damage. Results are expressed in arbitrary units (U.CARR), with 1 U.CARR corresponding to the oxidizing capacity equivalent to 0.08 mg/100 mL of hydrogen peroxide, rather than indicating a true H_2_O_2_ concentration (Costantini, 2016). Serum samples for 17β‐estradiol (E_2_), pregn‐4‐ene‐3,20‐dione (P_4_), and testosterone (T) were sent to an external laboratory (BML, Inc., Tokyo, Japan) and analyzed by chemiluminescent immunoassays. The lower limit of quantification for E2 was 10 pg/mL; values below this limit were treated as nonquantifiable (missing) and were excluded from E2‐specific analyses. For IL‐6, values outside the quantifiable range of the assay were treated as nonquantifiable (missing) and were excluded from IL‐6–specific analyses.

### 2.4 Statistical analysis

Normality was assessed using the Shapiro–Wilk test. Because IL‐6 concentrations were not normally distributed, values were transformed using the natural logarithm (ln) prior to analysis. For participant characteristics, between‐group differences in age and height were examined using one‐way analysis of variance (ANOVA), and the other participant characteristics were analyzed using linear mixed‐effects models. Each variable was treated as a dependent variable, with Group (males, MP, LP) included as a fixed effect and participant ID included as a random effect in the model.

Repeated‐measures data for IL‐6 and d‐ROMs were analyzed using linear mixed‐effects models to examine the main effects of Group and Time, as well as their interaction. The dependent variable was IL‐6 or d‐ROMs, and Group, Time, and the Group × Time interaction were included as fixed effects. Participant ID was included as a random effect, and Time was specified as a repeated measure within participants. Menstrual cycle phase was incorporated into the within‐subject covariance structure by specifying a Kronecker‐product unstructured covariance (UN_UN). To evaluate the influence of covariates, we constructed two models: a model without covariates (Model 1) and a model in which mean power output during exercise was added as a covariate (Model 2), and compared them. Using this approach, we examined whether the Group × Time interaction effect remained significant after controlling for differences in mean power output. Furthermore, carbohydrate oxidation during exercise and SMM were each added as covariates in separate models to examine their individual contributions.

Relationships of power output with carbohydrate oxidation and with SMM were examined using Pearson's correlation coefficients, whereas relationships of the change in IL‐6 from Pre to Post0 (ΔIL‐6) with carbohydrate oxidation and with power output were examined using Spearman's correlation coefficients.

For the one‐way ANOVA and the linear mixed‐effects models, *p* values for multiple comparisons were adjusted using the Bonferroni method. All statistical analyses were performed using SPSS version 30.0.0.0 (IBM, Armonk, NY, USA), and statistical significance was set at *p* < 0.05. Unless otherwise specified, data are presented as mean ± standard deviation (SD).

## 3 RESULTS

### 3.1 Participant characteristics

Table 1 presents the mean ± SD values for each variable. Of the 14 females who participated, one was excluded from the analyses at the discretion of a physician because her E_2_ concentration was abnormally high during the MP trial. Thus, 13 females were included in the analyses. For these 13 females, the menstrual cycle length was 31.1 ± 4.9 days. Measurements in the MP were performed on day 3.3 ± 0.7 of the cycle, and LP measurements were performed on day 22.5 ± 3.4. All 15 males were included in the analyses. Among the 13 females included, IL‐6 data were available for *n* = 12 because one female participant had IL‐6 concentrations outside the quantifiable range of the assay in both the MP and LP. Carbohydrate oxidation analyses in females were conducted with *n* = 12 in both the MP and LP because gas analysis data were missing for one woman during the LP test due to equipment failure, and the corresponding MP data for that participant were also excluded to maintain paired comparisons. In males, E2 data were available for *n* = 9 because values below the assay lower limit of quantification (10 pg/mL) were nonquantifiable.

**TABLE 1:** Participant characteristics and exercise variables.

	Female		Male	*p* Value		
	MP	LP		Phase	Sex	Sex
				(MP vs. LP)	(MP vs. male)	(LP vs. male)
Age	25.7 ± 2.8 (23–32)		26.5 ± 2.9 (23–33)	–	0.451	
Height (cm)	160.3 ± 5.9		172.4 ± 7.0	–	<0.001	
Weight (kg)	54.4 ± 3.0	54.6 ± 3.0	70.4 ± 12.0	1.000	<0.001	<0.001
SMM (kg)	21.0 ± 1.5	21.0 ± 1.5	31.89 ± 5.15	1.000	<0.001	<0.001
Power output (W)	42.8 ± 11.8	44.0 ± 14.1	78.8 ± 26.9	0.956	<0.001	<0.001
RPE Reg	12.2 ± 1.7	12.5 ± 1.3	12.2 ± 1.7	1.000	1.000	0.507
RPE Breath	11.24 ± 1.8	11.53 ± 1.4	10.0 ± 2.0	0.527	0.262	0.114
Carbohydrate oxidation (g/min)	0.62 ± 0.12	0.69 ± 0.23	1.09 ± 0.27	0.670	<0.001	<0.001
E_2_ (pg/mL)	37.7 ± 19.5	168.4 ± 85.3	17.5 ± 5.8	<0.001	–	–
P_4_ (ng/mL)	0.3 ± 0.1	9.0 ± 6.8	0.3 ± 0.1	<0.001	1.000	<0.001
T (pg/dL)	34.8 ± 8.8	38.2 ± 9.2	698.1 ± 178.9	0.351	<0.001	<0.001

*Note*: Data are presented as mean ± SD. Sample size: E2 (males), *n* = 9; carbohydrate oxidation (MP and LP), *n* = 12.

Abbreviations: E2, 17β‐estradiol; LP, luteal phase; MP, menstrual phase; P4, pregn‐4‐ene‐3,20‐dione; RPE, rating of perceived exertion; SMM, skeletal muscle mass; T, testosterone.

### 3.2 Exercise‐induced IL‐6 responses: Unadjusted and adjusted for power output

**FIGURE 1:**
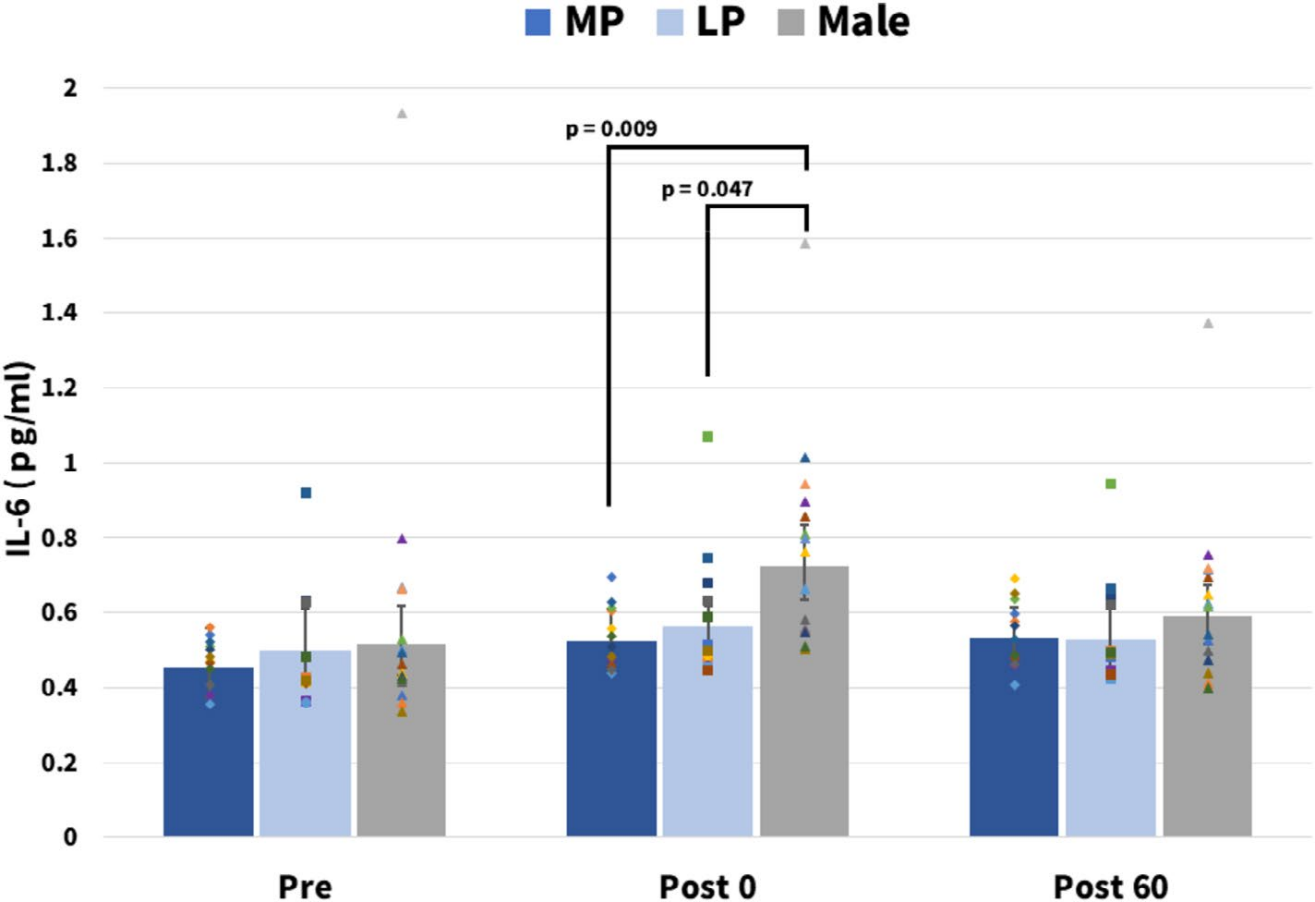
Unadjusted exercise‐induced changes in IL‐6 in males and females (Model 1). Estimated marginal means of IL‐6 derived from linear mixed‐effects Model 1 (without covariates) were back‐transformed from the log scale using the exponential function and are presented in the original units. Error bars represent 95% confidence intervals. These differences became nonsignificant in Model 2, in which mean power output during exercise was included as a covariate. Sample size: MP and LP, *n* = 12. LP, luteal phase; MP, menstrual phase.

Figure 1 shows IL‐6 concentrations at Pre, Post0, and Post60 expressed as back‐transformed from the log scale using the exponential function and are presented in the original units. Error bars represent 95% confidence intervals.

In Model 1 of the linear mixed‐effects model, which did not include covariates, a significant group × time interaction was observed (*p* < 0.001). Post hoc comparisons were performed to examine temporal changes within each group and differences between groups at each time point. IL‐6 increased significantly from Pre to Post0 in all groups. At Post0, males had significantly higher IL‐6 concentrations than females in both the MP and LP (Figure 1). No group differences were observed at Pre or Post60.

Next, because IL‐6 responses depend on workload such as the amount of muscle recruited and metabolic demand, and because in the present study differences between males and females were observed in mean power output during exercise, which was used as an index reflecting these factors (Table 1), we constructed Model 2 including mean power output during exercise as a covariate. In Model 2, the Group × Time interaction remained significant; however, the Post0 differences between males and females (MP and LP) that were significant in Model 1 were no longer statistically significant in the covariate‐adjusted model (Model 2).

After adjusting for carbohydrate oxidation, the Post0 differences between males and females (MP and LP) that were significant in Model 1 were no longer statistically significant, as with adjustment for mean power output. In contrast, when SMM was included as a covariate, the Post0 differences between males and females (MP and LP) remained statistically significant, consistent with Model 1.

### 3.3 Exercise‐induced d‐ROMs responses

**FIGURE 2:**
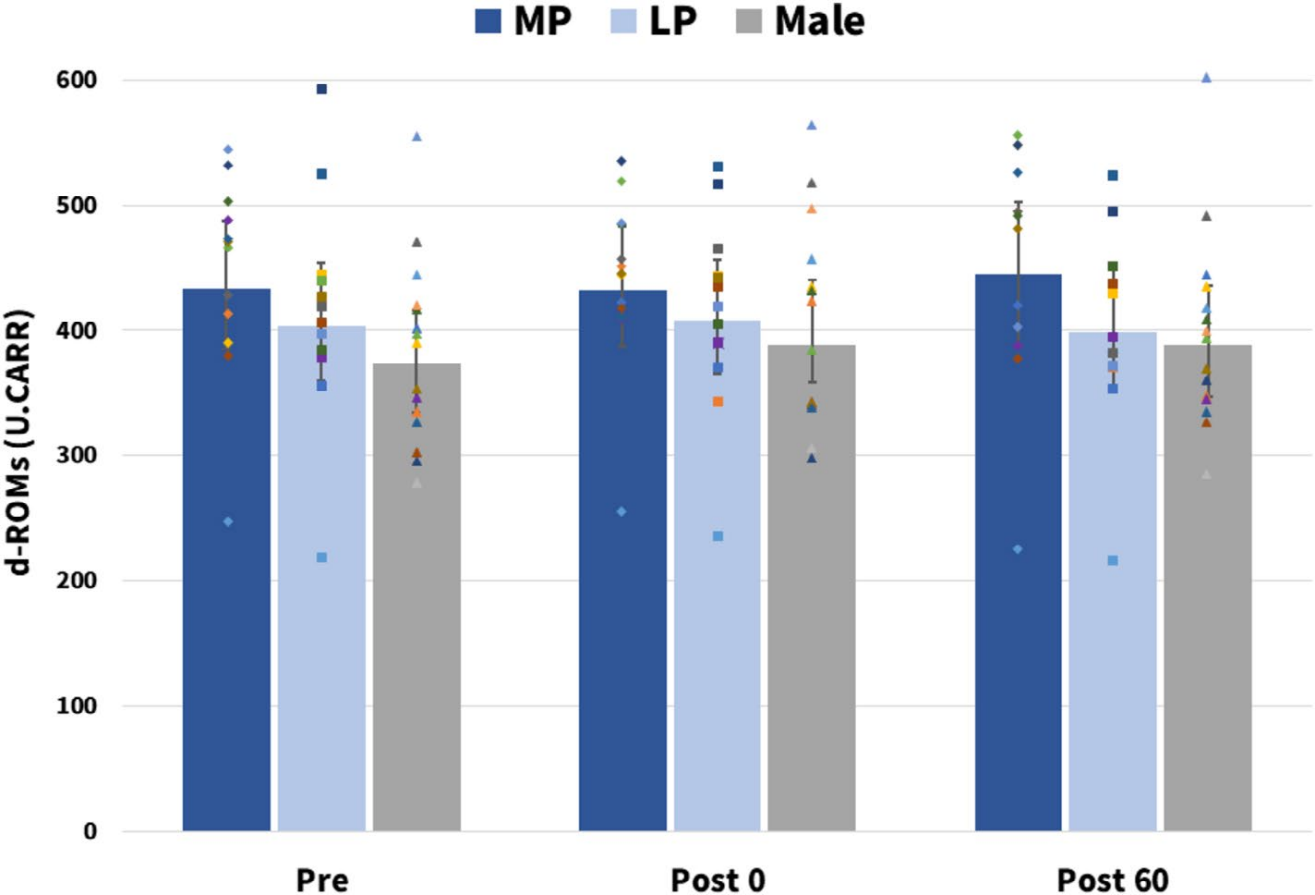
Exercise‐induced changes in d‐ROMs in males and females. The estimated marginal means of d‐ROMs derived from the linear mixed‐effects model are shown. Error bars represent 95% confidence intervals. LP, luteal phase; MP, menstrual phase.

Figure 2 shows the estimated marginal means of d‐ROMs at Pre, Post0, and Post60, derived from the linear mixed‐effects model, together with 95% confidence intervals. In the analysis, neither the main effect of Group (males, MP, LP) nor that of Time was significant, and no Group × Time interaction was observed. Thus, in this study, a single bout of moderate‐intensity aerobic exercise induced only small changes in d‐ROMs, a marker of oxidative stress, and the time course of these changes did not differ among groups.

### 3.4 Relationships of power output with carbohydrate oxidation and SMM

**FIGURE 3:**
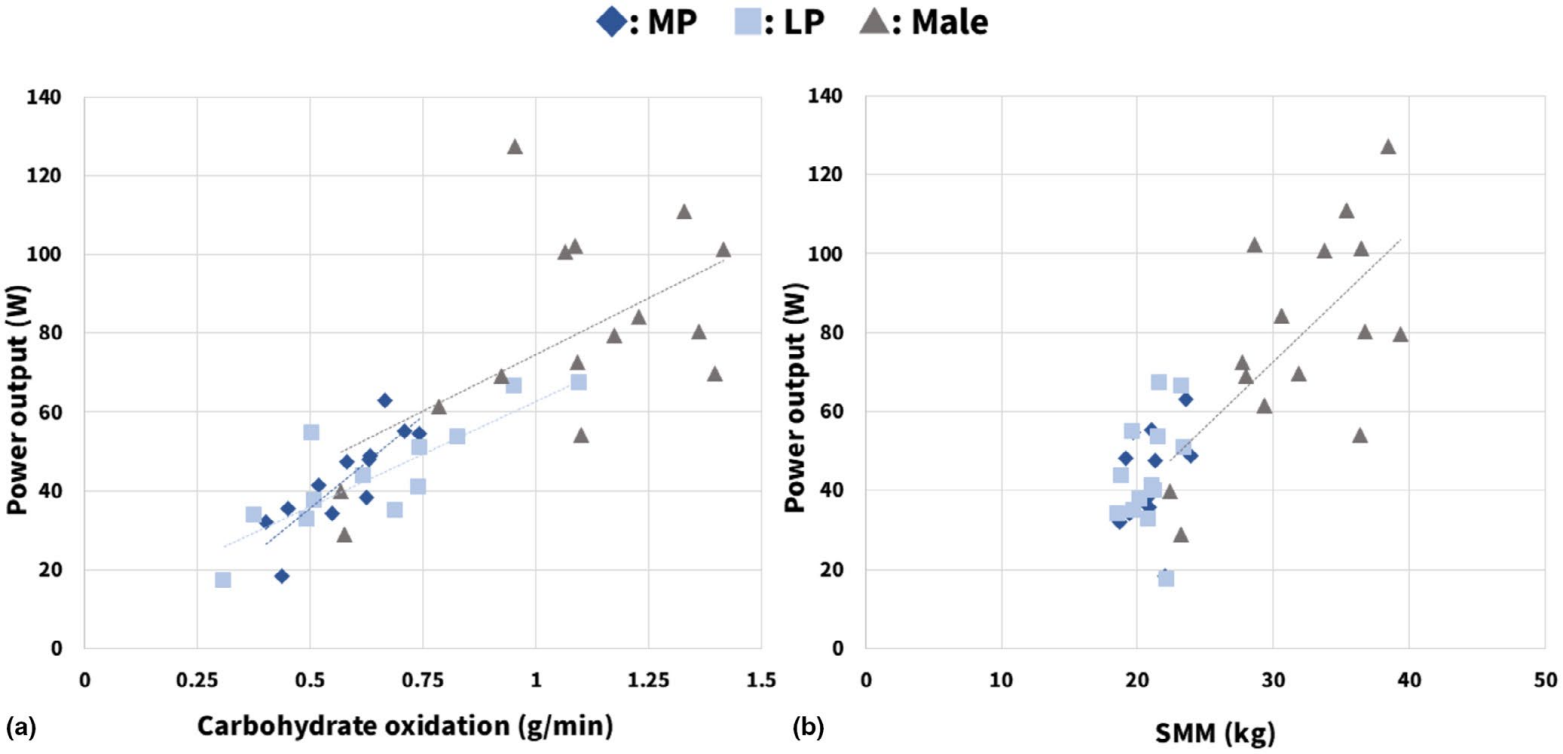
Relationships between power output, carbohydrate oxidation, and SMM. (a) Relationship between power output and carbohydrate oxidation. Significant moderate to strong positive correlations were observed in all groups. (b) Relationship between power output and SMM. A significant moderate positive correlation was observed only in males. In both panels, dotted regression lines indicate statistically significant relationships. Sample size: (a) (MP and LP), *n* = 12. MP, menstrual phase; LP, luteal phase; SMM, skeletal muscle mass.

Figure 3a shows the relationship between power output and carbohydrate oxidation. The regression equations for each group were as follows: MP, y = 93.964x − 11.293 (*r* = 0.839, *p* < 0.001); LP, y = 53.246x + 9.4194 (*r* = 0.845, *p* < 0.001); males, y = 57.293x + 17.47 (*r* = 0.682, *p* = 0.005). All groups showed moderate to strong positive correlations.

Figure 3b shows the relationship between power output and SMM. The regression equations for each group were as follows: MP, y = 2.0901x − 0.9905 (*r* = 0.278, *p* = 0.357); LP, y = 3.0736x − 20.464 (*r* = 0.332, *p* = 0.268); males, y = 3.2878x − 26.04 (r = 0.653, *p* = 0.008), indicating that a significant moderate positive correlation was observed only in males.

### 3.5 Relationships of ΔIL‐6 with power output and carbohydrate oxidation

**FIGURE 4:**
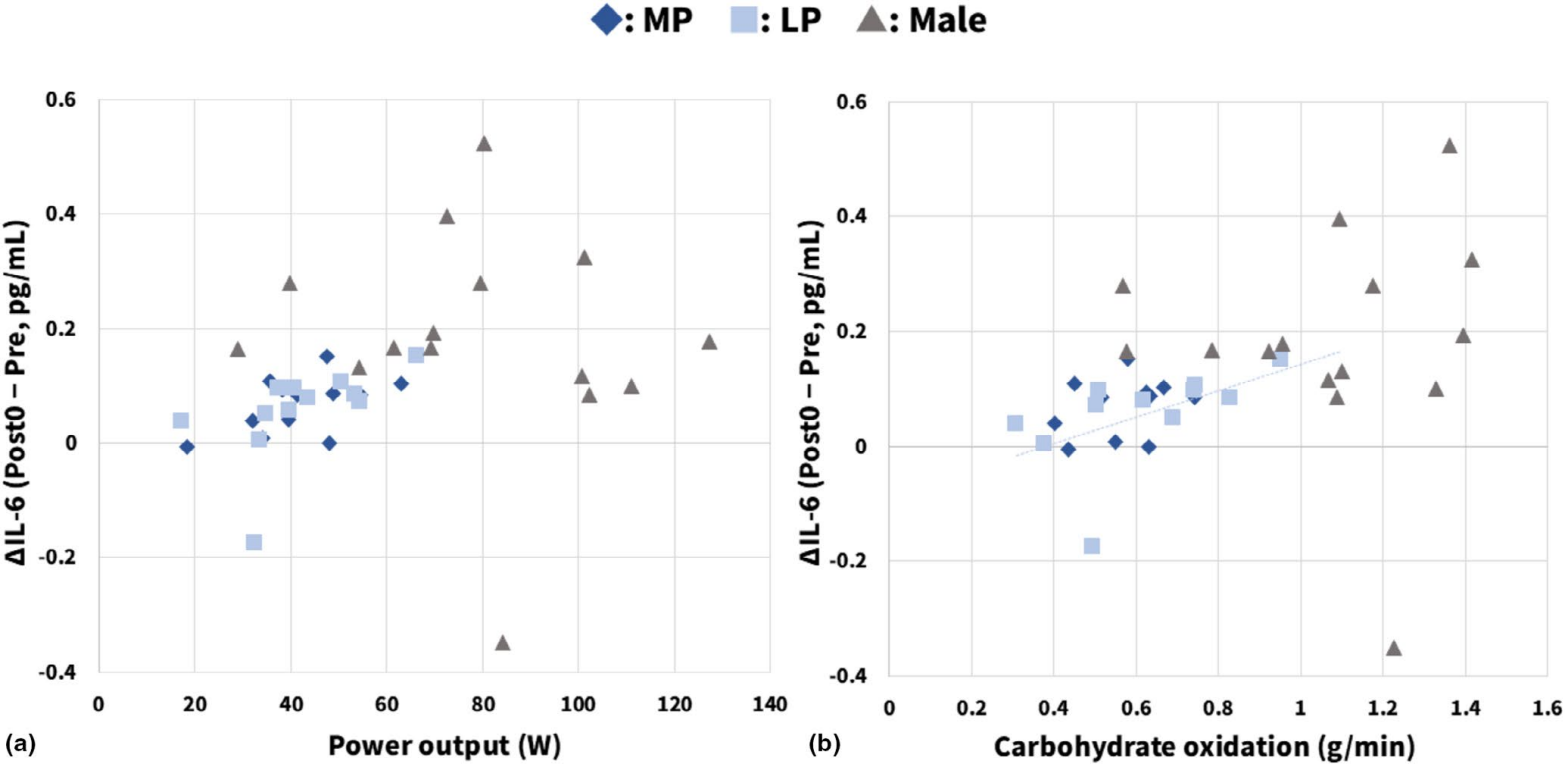
Relationships between changes in ΔIL‐6, power output, and carbohydrate oxidation. (a) Relationship between ΔIL‐6 and power output. No significant correlations were observed in any group. (b) Relationship between ΔIL‐6 and carbohydrate oxidation. A strong positive correlation was observed only in the LP. In both panels, dotted regression lines indicate statistically significant relationships. Sample size: (a) *n* = 12 (MP and LP); (b) (MP and LP), *n* = 11. LP, luteal phase; MP, menstrual phase.

The relationship between ΔIL‐6 and power output during exercise is shown in Figure 4a. The regression equations for each group were as follows: MP, y = 0.0022x − 0.0263 (ρ = 0.441, *p* = 0.152); LP, y = 0.0034x − 0.0892 (ρ = 0.427, *p* = 0.167); males, y = −0.0008x + 0.249 (ρ = −0.171, *p* = 0.541), and no significant correlations were observed in any of the groups.

The relationship between ΔIL‐6 and carbohydrate oxidation during exercise is shown in Figure 4b. The regression equations for each group were as follows: MP, y = 0.1394x − 0.0103 (ρ = 0.291, *p* = 0.385); LP, y = 0.2309x − 0.0882 (ρ = 0.800, *p* = 0.003); males, y = 0.0265x + 0.1555 (ρ = 0.186, *p* = 0.508). A significant correlation was observed only in the LP, and not in the MP or in males.

## 4 DISCUSSION

This study examined differences between males and females in IL‐6 responses to moderate‐intensity aerobic exercise performed at the same relative intensity and explored whether these differences were influenced by skeletal muscle mass (SMM) and exercise‐related metabolic demand. At Post0, IL‐6 levels were higher in males than in females. However, this difference was no longer statistically significant after adjusting for power output. Power output was positively associated with carbohydrate oxidation during exercise; the difference in IL‐6 levels was no longer statistically significant after adjustment for carbohydrate oxidation. These findings suggest that the observed difference in IL‐6 response was primarily attributable to differences in metabolic demand associated with higher absolute workload, rather than intrinsic biological differences between males and females. In contrast, the d‐ROMs response to moderate‐intensity aerobic exercise was small across groups, and no clear between‐group differences were observed. This study highlights the need for more precise analysis and interpretation in research investigating differences between males and females in exercise physiology.

IL‐6 is one of the cytokines whose blood concentration increases markedly during exercise (Jouffroy et al., 2022; Suzuki et al., 2000, 2003). In the present study, IL‐6 increased significantly from Pre to Post0 in all groups, and this increase was significantly greater in males than in females (Figure 1). The amount of IL‐6 produced during exercise is known to be influenced by factors such as exercise duration, exercise intensity, the amount of recruited muscle mass, and the extent of carbohydrate utilization (Febbraio & Pedersen, 2002; Helge et al., 2003; Holmes et al., 2004; Steensberg et al., 2001). Accordingly, we used the power output over the 60 min cycling exercise, which was considered to comprehensively reflect these factors (exercise intensity, recruited muscle mass, and carbohydrate metabolism), as a representative index and entered it as a covariate in the linear mixed‐effects model. As a result, the differences between males and females in IL‐6 responses observed at Post0—namely, the greater response in males than in females in both the MP and LP groups—were no longer statistically significant in Model 2 (adjusted for power output). This indicates that the differences between males and females in the IL‐6 response observed under the conditions of this study were related to the difference in power output.

Subsequently, to assess the extent to which mean power output was related to carbohydrate oxidation and SMM, we performed correlation analyses within each group between mean power output and carbohydrate oxidation during exercise and SMM. Power output showed significant, moderate to strong positive correlations with carbohydrate oxidation in all groups (MP, LP, and males), and data points for each group were distributed along nearly the same regression line (Figure 3a). In contrast, for the relationship between power output and SMM, a moderate positive correlation was observed only in males, whereas no significant correlation was found in females (Figure 3b). Based on these findings, we further added carbohydrate oxidation during exercise and SMM individually as covariates to the linear mixed model, as with power output. Only adjustment for carbohydrate oxidation eliminated the differences between males and females at Post0, mirroring the effect of including power output. Therefore, the observed differences in IL‐6 response were thought to reflect differences in carbohydrate metabolism during exercise more strongly than SMM, at least under the conditions of this study.

Furthermore, to examine the relationships between ΔIL‐6 and power output and carbohydrate oxidation during exercise, we analyzed the correlations between these variables within each group. ΔIL‐6 showed no significant correlations with power output in any group (Figure 4a), whereas it was significantly positively correlated with carbohydrate oxidation during exercise only in females in the LP group (Figure 4b). Although the influence of sample size must be taken into account, these findings suggest that, at least under the conditions of the present study, the magnitude of carbohydrate oxidation and power output during exercise in each group may not exhibit a simple dose–response relationship with IL‐6 responses. At the level of individual participants, baseline metabolic status and pre‐exercise muscle glycogen levels are likely to differ. In addition, it is known to involve complex contributions from multiple tissues and cell types, including immune cells, adipose tissue, the central nervous system, and skeletal muscle fibers (Lyngsø et al., 2002; Nybo et al., 2002; Tominaga et al., 2019). These multiple sources of inter‐individual variability may have contributed to the fact that IL‐6 responses could not be captured as a simple dose–response relationship.

In the moderate‐intensity aerobic exercise bout used in this study, d‐ROMs did not show significant time‐dependent changes (Figure 2). Previous studies have demonstrated that d‐ROMs increase significantly shortly after exercise during high‐intensity interval training or intense endurance exercise performed at intensities substantially exceeding the anaerobic threshold (Kon et al., 2021; Takahashi et al., 2012). Furthermore, d‐ROMs have been reported to change following high‐load endurance competitions such as races (Sugama et al., 2015). At the relatively moderate aerobic exercise intensity used in this study, the oxidative stimulus was likely insufficient to markedly alter systemic total hydroperoxide levels. Moreover, d‐ROMs are known to be influenced by numerous factors, including daily lifestyle habits, chronic inflammatory states, and the activity of endogenous antioxidant systems (Hirose et al., 2009; Ishizaka et al., 2013; Schöttker et al., 2021). Such variability in these background factors may have limited our ability to detect statistically significant exercise‐induced changes in this study. To more clearly capture sex differences in oxidative stress responses to exercise in the future, it would be useful to employ protocols expected to induce a rapid and pronounced rise in oxidative stress (e.g., higher‐intensity and/or longer‐duration exercise) and to assess oxidative stress using d‐ROMs in combination with more specific oxidative stress markers.

In the present study, we focused on differences between males and females in the acute responses to a single bout of exercise. The present findings indicate that the observed difference in IL‐6 response between males and females was associated with differences in power output under conditions where relative intensity was matched and with accompanying differences in carbohydrate metabolism. Many previous exercise physiology studies examining sex differences have not adequately accounted for differences in the workload imposed on males and females (Ansdell et al., 2020; Aragón‐Vela et al., 2021; Benini et al., 2015; Edwards et al., 2006; Landen et al., 2023; Timmons et al., 2005). Thus, at least part of the previously reported sex differences may reflect differences in the workload imposed between males and females. However, in real‐world training settings, males and females often differ substantially in SMM and maximal oxygen uptake. As a result, even when exercise is prescribed at the same relative intensity (e.g., %VO_2max_ or RPE), the absolute workload does not necessarily match between males and females.

Therefore, when exercise guidelines and training recommendations that are largely based on data from young males (Mann et al., 2013; Pandit et al., 2023) are applied to females without sufficient consideration of differences between males and females in external workload, beneficial molecular responses may not be adequately elicited, potentially leading to differences in training adaptations and long‐term health benefits. Furthermore, in recent years, the development of exercise mimetics as pharmacological alternatives to physical exercise has progressed (Gubert & Hannan, 2021; Hawley et al., 2021). However, it will be essential to correctly understand the diversity of molecular responses arising from differences in sex, body size, and metabolism and to individualize prescriptions and interventions accordingly. The present study provides a novel framework for investigating sex differences in exercise physiology by emphasizing the importance of accounting for absolute workload. This approach may contribute to optimizing exercise prescription according to individual characteristics and to improving the precision of sex‐specific medical and scientific research.

In this study, we took into account fluctuations in the female endocrine environment that may influence molecular responses by repeatedly measuring the same female participants in two phases of the menstrual cycle and including these data in the analyses. Examining differences between males and females while partially controlling for intraindividual differences in hormonal milieu in this way is an important strength of the study and provides useful insights for developing future training strategies that consider differences between males and females and menstrual cycle phase. However, several limitations should be noted. First, the participants in this study were limited to young, generally healthy adults, and caution is required when generalizing these findings to other populations. Second, the present findings are based on statistical associations used to infer relationships between external workload and molecular responses; further studies are needed to elucidate the underlying mechanisms. Third, ovulation testing was not performed to confirm menstrual cycle phase in female participants. In addition, measurements were limited to two time points—MP and LP—and hormone levels in the LP showed some variability, which should also be taken into account when interpreting the results.

## AUTHOR CONTRIBUTIONS

**Ena Yoshida:** Conceptualization; data curation; formal analysis; funding acquisition; investigation; methodology; project administration; resources; software; supervision; validation; visualization. **Harumi Hayashida:** Funding acquisition; supervision. **Tung Lin Lu:** Investigation; validation. **Yasuhiro Seki:** Resources. **Katsuhiko Suzuki:** Investigation; resources; supervision.

## FUNDING INFORMATION

Ena Yoshida, JST SPRING, Grant Number: JPMJSP2128; Harumi Hayashida, JSPS KAKENHI, Grant Number: 25K14707.

## CONFLICT OF INTEREST STATEMENT

The authors have no conflicts of interest, financial or otherwise, to disclose.

## Data Availability

The datasets generated and/or analyzed during the current study are not publicly available due to ethical and privacy restrictions but are available from the corresponding author on reasonable request.
